# Transcriptome Sequencing Revealed an Inhibitory Mechanism of *Aspergillus flavus* Asexual Development and Aflatoxin Metabolism by Soy-Fermenting Non-Aflatoxigenic *Aspergillus*

**DOI:** 10.3390/ijms21196994

**Published:** 2020-09-23

**Authors:** Kunlong Yang, Qingru Geng, Fengqin Song, Xiaona He, Tianran Hu, Shihua Wang, Jun Tian

**Affiliations:** 1 School of Life Science, Jiangsu Normal University, Xuzhou 221116, China; ykl_long@yeah.net (K.Y.); qingru950504@163.com (Q.G.); songfengqin@jsnu.edu.cn (F.S.); xiaona_he@163.com (X.H.); 2 Key Laboratory of Pathogenic Fungi and Mycotoxins of Fujian Province, School of Life Sciences, Fujian Agriculture and Forestry University, Fuzhou 350002, China; hutianran77@yeah.net

**Keywords:** *Aspergillus flavus*, transcriptome, aflatoxin, conidiation, RNA-seq

## Abstract

Aflatoxins (AFs) have always been regarded as the most effective carcinogens, posing a great threat to agriculture, food safety, and human health. *Aspergillus flavus* is the major producer of aflatoxin contamination in crops. The prevention and control of *A. flavus* and aflatoxin continues to be a global problem. In this study, we demonstrated that the cell-free culture filtrate of *Aspergillus oryzae* and a non-aflatoxigenic *A. flavus* can effectively inhibit the production of AFB1 and the growth and reproduction of *A. flavus*, indicating that both of the non-aflatoxigenic *Aspergillus* strains secrete inhibitory compounds. Further transcriptome sequencing was performed to analyze the inhibitory mechanism of *A. flavus* treated with fermenting cultures, and the results revealed that genes involved in the AF biosynthesis pathway and other biosynthetic gene clusters were significantly downregulated, which might be caused by the reduced expression of specific regulators, such as AflS, FarB, and MtfA. The WGCNA results further revealed that genes involved in the TCA cycle and glycolysis were potentially involved in aflatoxin biosynthesis. Our comparative transcriptomics also revealed that two conidia transcriptional factors, *brlA* and *abaA*, were found to be significantly downregulated, which might lead to the downregulation of conidiation-specific genes, such as the conidial hydrophobins genes *rodA* and *rodB*. In summary, our research provides new insights for the molecular mechanism of controlling AF synthesis to control the proliferation of *A. flavus* and AF pollution.

## 1. Introduction

*Aspergillus flavus* is a common saprophytic fungus that contaminates many important seed crops, including peanuts, corn, and pistachios [1,2]. The contamination of *A. flavus* causes huge economic losses to agricultural production across the world. This fungus is also an opportunistic pathogen for immunocompromised patients, which is the second most common cause of aspergillosis after *A. fumigatus* [3]. *A. flavus* is notorious for its production of one of the most carcinogenic mycotoxins, aflatoxin [4], which has an extensive relationship with liver cancer [5]. Therefore, it is important to develop effective and safe approaches to control this fungus and inhibit the production of aflatoxins for food safety and human health.

To prevent the contamination of *A. flavus* and aflatoxins on agricultural products and foodstuffs, which occur mostly at pre- and post-harvest periods, farmers and manufacturers have kept them at conditions of low temperature and low humidity. Numerous strategies, including physical control (e.g., heat and ultraviolet radiation), chemical control (e.g., using natural preservative) [6,7,8], and biological control (e.g., microbial competition), have been applied to combat this fungus and control aflatoxin production. Due to the efficient elimination of mycotoxins and to the desire for a safe food supply, biological control represents an attractive choice. A range of microbes, such as bacteria [9,10,11], yeast, and fungi (e.g., nontoxigenic *Aspergillus*), have been used for biocontrol aflatoxigenic *Aspergillus* [12]. Several biological control strategies to reduce AF contamination have been developed, including the use of non-toxic *A. flavus* and other fungi to inhibit aflatoxin synthesis [13]. Shakeel et al. reported that the bacterium *Streptomyces yanglinensis* 3-10 was able to produce antifungal substances that reduce the postharvest decay of peanut kernels by inhibiting mycelia growth of *A. flavus* and AFB_1_ production [14]. A previous study reported that spreading non-toxic *A. parasiticus* strains on peanut-growing soil could reduce the AF content in edible peanuts by 83% to 98% [15]. Fungi, such as white-rot fungus, *Rhizopus pseudomonas*, and *A. niger*, have been utilized to control aflatoxin biosynthesis [16]. Of these filamentous fungi, non-toxigenic aspergilli used for fermented foods, such as *A*. *oryzae* and *A*. *niger*, could be the best prospect for the sake of their safety. 

Transcriptome sequencing has been utilized for a better understanding of the inhibitory mechanisms of antagonistic microbes against *A. flavus*. A biocontrol yeast against *A. flavus* called *Wickerhamomyces anomalus* was found to inhibit AF production by repressing the activation of the AF biosynthetic pathway cluster [17]. The extracts from the medicinal plant *Micromeria graeca* were also reported to restrict AF biosynthesis, without an effect on fungal growth, by the downregulation of *aflR* and *aflS*, two activators of the AF gene cluster, and the overexpression of two major global regulators, *veA* and *mtfA* [18]. Zhao et al. previously revealed the inhibitory effects of *Lactobacillus plantarum* on AF biosynthesis using transcriptomic analysis, which showed that the transcriptional levels of genes involved in the AF biosynthetic process were significantly downregulated, while genes related to the synthesis and organization of cell wall polysaccharides were upregulated, which might be related to the impaired effects of *L. plantarum* on the cellular structure of fungal tissue [19].

In this study, we used two non-aflatoxigenic *Aspergillus* strains (*A. oryzae* and an MAPK kinase SskB null mutant of *A. flavus* named TSJ-1 that fails to produce aflatoxins) to control the growth and development of the aflatoxin synthesis of *A. flavus*. We found that, in a co-culture system with *A. oryzae* and a non-aflatoxigenic *A. flavus* generated genetically, the production of AFB1 was dramatically inhibited. Furthermore, the cell-free culture filtrate of these two non-aflatoxigenic *Aspergillus* strains was enabled and effectively blocked AF biosynthesis and *A. flavus* development as well. Further comparative transcriptome sequencing was applied to reveal the inhibition mechanism of AF metabolism and *A. flavus* development by *A. oryzae*.

## 2. Results

### 2.1. The Effect of Non-Aflatoxigenic Aspergilli on A. flavus Growth and Aflatoxin B1 (AFB1) Production by Co-Cultivation with A. flavus

**Figure 1:**
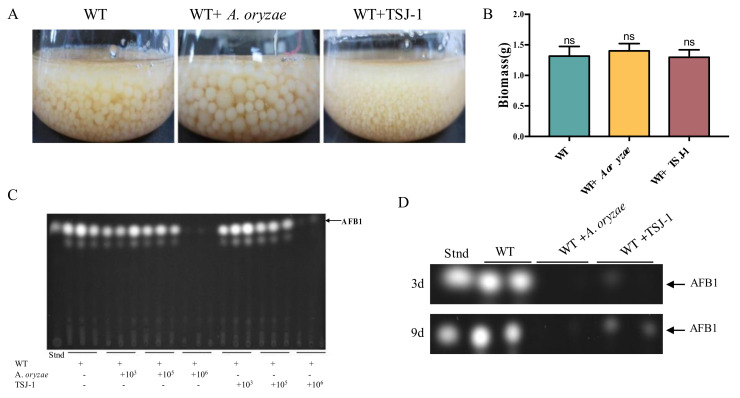
The growth phenotype and aflatoxin B1 production of *A. flavus* when co-cultured with non-aflatoxigenic *Aspergillus* strains. (**A**) The growth phenotype of *A. flavus* when co-cultured with *A. oryzae* or TSJ-1 strain in liquid-shake YES (yeast extract and sucrose) medium at 30 °C for 9 days. (**B**) The total mycelium dry weight of *A. flavus* co-cultured with the indicated strains. ns indicates no significance. (**C**) Detection result of aflatoxins production after *A. flavus* was co-cultured with different concentrations of non-aflatoxigenic *Aspergillus* strains’ spores at 30 °C for 4 days. In total, 1 mL of 10^6^ *A. flavus* spores was added to a 50-mL YES medium. (**D**) TLC results of AFB1 production when *A. flavus* was co-cultured with the indicated strains in a liquid-shake YES medium for 3 and 9 days, respectively.

To test whether the non-aflatoxigenic *Aspergillus* strains affect *A. flavus* growth and AFB1 accumulation under a co-culture system, a total number of 10^6^ conidia of *A. oryzae* RIB40/TSJ-1 were co-cultivated with an equal amount of *A. flavus* NRRL3357 spores in 50 mL of YES medium for 9 days. The results showed that the total biomass of the co-culture system displayed no difference compared to the wild type (Figure 1B), while *A. flavus* co-cultivated with *A. oryzae* was greater in mycelium pellets compared with the wild-type control, and smaller ones were found when co-cultured with TSJ-1 (Figure 1A). The experiment that *A. flavus* co-cultivated with different concentrations of spores of *A. oryzae*/TSJ-1 demonstrated that an increasing conidia amount to 10^6^ of the non-aflatoxigenic *Aspergillus* strains could apparently block AFB1 accumulation (Figure 1C). The AFB1 production in the co-culture medium with an equal amount of *Aspergillus* spores (10^6^) was assayed after 3 days and after 9 days of incubation as well, which showed that AFB1 production was decreased after 9 days of incubation in the wild-type control compared to the 3 days of incubation (Figure 1D), and AFB1 production was dramatically inhibited in the co-culture system at both of these two time points, while a detectable level of AFB1 could still be found in the TSJ-1 co-cultures (Figure 1D).

### 2.2. Cell-Free Concentrated Filtrates of Non-Aflatoxigenic Aspergilli inhibit A. flavus Asexual Development and AFB1 Accumulation

**Figure 2:**
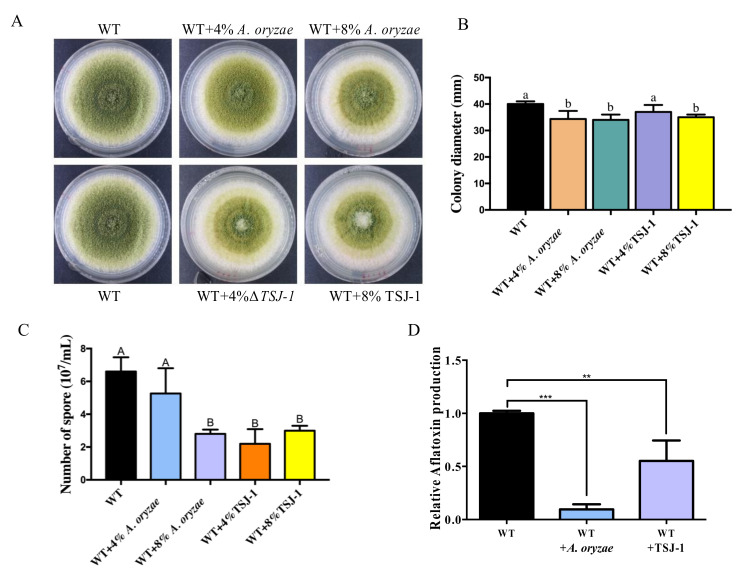
Effect of the culture filtrate of non-aflatoxigenic *Aspergillus* strains on *A. flavus* reproduction and AFB1 production. (**A**) Growth phenotype of the wild-type *A. flavus* strain on PDA medium treated with different concentrations of the culture filtrate of the indicated strains for 4 days. (**B**) Colony diameter of *A. flavus* in (**A**). (**C**) Conidia production of *A. flavus* in (**A**). (**D**) The effect of the culture filtrate of the indicated stains on AFB1 production in a liquid-shake YES medium for 4 days. A total of 10^6^ *A. flavus* spores were added to a 50-mL YES medium with 8% of the culture filtrate of the indicated strains. Different uppercase letters above the bars represent significantly different values (*p* < 0.01), while different lowercase letters above the bars represent *p* < 0.05. ** statistically significant when compared to control, *p* < 0.01; *** statistically significant when compared to control, *p* < 0.001.

To determine the effect of the culture filtrate of *A. oryza* and TSJ-1 on *A. flavus* development, *A. flavus* was inoculated on solidified PDA plates containing with or without different concentrations of the concentrated filtrates. An inhibitory growth of wild-type *A. flavus* was found within the treatment of *A. oryzae* culture filtrates and 8% of the TSJ-1 culture filtrate (Figure 2A,B). The result also showed that a significant decrease in conidia pigmentation occurred in the treatment of *A. oryzae*/TSJ-1 culture filtrates when compared to the wild-type control (Figure 2A). Further determination of conidiation showed that *A. flavus* was significantly reduced in conidia production in the treatment of TSJ-1 culture filtrates and 8% of *A. oryzae* culture filtrate (Figure 2C).

To assay its influence on AFB1 biosynthesis, *A. flavus* NRRL3357 was grown in YES media supplemented with 8% of the concentrated filtrates. The results showed that *A. oryzae* culture filtrates were found to be highly effective in inhibiting AFB1 production with an inhibition rate of 90.43%, while the inhibition rate for the TSJ-1 filtrates was 44.8% (Figure 2D). Taken together, these data demonstrated that filtrates of non-aflatoxigenic aspergilli have inhibitory effects on *A. flavus* asexual conidiation and AFB1 production.

### 2.3. RNA-seq Analysis of A. flavus by the Treatment of A. oryzae Culture Filtrate

**Figure 3:**
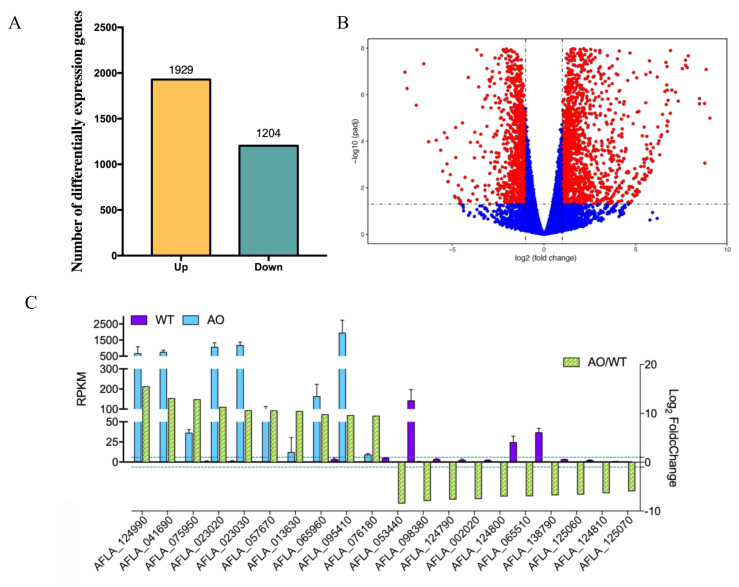
Analysis of differentially expressed genes (DEGs) in *A. flavus* treatment with *A. oryzae* Rib40 cell-free culture filtrate. (**A**) Number of genes showing upregulated and downregulated expression of WT strain cultured in PDB with/without *A. oryzae* cell-free culture filtrate treatment. (**B**) Volcano plots of the DEGs in the wild-type strain with or without *A. oryzae* culture filtrate treatment. The blue spots inside the grey dash lines indicate the non-significant DEGs. (**C**) Relative expression levels of the top 10 upregulated/downregulated DEGs. Left Y axis indicates the RPKM values of the selected DEGs in WT (*A. flavus* wild type without any treatment) and AO (*A. flavus* wild type with *A. oryzae* cell-free culture filtrate treatment); Right Y axis presents the log2 FoldChange of the selected DEGs in AO when compared to WT. The dashed line indicates |log2 FoldChange| = 1.

To reveal the regulatory molecular mechanism of non-aflatoxigenic aspergilli culture filtrate against *A. flavus*, RNA-seq analysis was carried out. Here, since the *A. oryzae* RIB40 WT strain was found more effective in inhibiting AFB1 production, *A. flavus* vegetatively grown in the presence or absence of *A. oryzae* culture filtrate was further sampled and analyzed using RNA-seq. A total of 38.52 Gb of clean base of 6 cDNA libraries were gained, and more than 5.7 Gb clean base for each biological repeat were obtained (Table S1). The Q20 and Q30 for each biological repeat were over 90% and 96%, respectively (Table S1). RNA sequences of *A. flavus* NRRL 3357 were further processed by quantile normalization of counts per million of counts uniquely mapping to each gene model, and only unique reads were used for the calculation of normalized gene expression as RPKM (reads per kilobase of transcript per million mapped reads). To characterize gene sets in response to the treatment of *A. oryzae* filtrates, a Venn diagram (Figure 3A) and volcano plots (Figure 3B) according to log10 of padj (*y*-axis) and log2 of fold change (*x*-axis) were used to visualize the common differentially expressed genes (DEGs), which indicated that more than 3100 DEGs (including 1204 downregulated and 1929 upregulated genes) were found in response to the treatment of *A. oryzae* filtrates (Figure 3A, Table S3). Analysis of the top 10 upregulated/downregulated DEGs showed that a gene predicted to encode a hypothetical FAD/NAD(P)-binding protein (AFLA_124990), a putative cytochrome P450 oxidoreductase GliC-like gene (AFLA_023030), and the fucose-specific lectin gene *fleA* (AFLA_065960) was one of the most highly expressed genes, increased by more than 800-fold compared with control *A. flavus*, while the conidial hydrophobin gene *rodA* (AFLA_098380), a putative spherulin 4-like cell surface protein coding gene (AFLA_002020), and a putative efflux pump antibiotic resistance protein coding gene (AFLA_125070) were one of the most downregulated genes, decreased by more than 50-fold compared to the wild-type control (Figure 3C). Additionally, we found that most of the molecular chaperone (heat shock proteins) and two alternative oxidases were transcriptionally activated in response to the treatment of *A. oryzae* filtrates (Figure S1B), and, intriguingly, most of the G protein-coupled receptors were found to be downregulated (Figure S1A).

### 2.4. GO Enrichment and KEGG Pathways Analysis of DEGs

**Figure 4:**
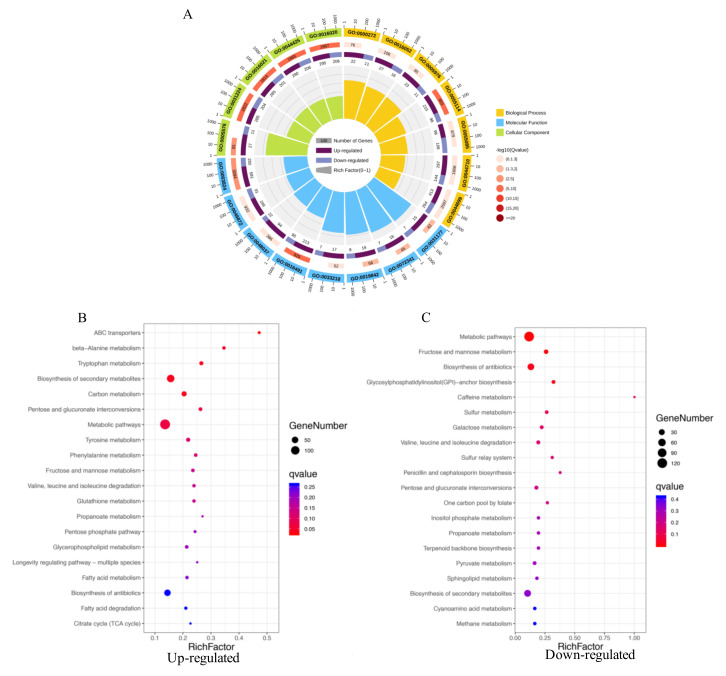
Enriched GO terms and KEGG pathways of differentially expressed genes (DEGs). (**A**) Enriched GO terms of DEGs. The numbers on the periphery represent a ruler for gene numbers, while the numbers in boxes indicate the threshold value of the “-log qvalue”. (**B**) Enriched KEGG pathways of the 1929 upregulated DEGs and (**C**) the 1204 downregulated DEGs that show the top 20 pathways of KEGG enrichment analysis.

The DEGs were further utilized for GO term analysis, including biological processes, cellular components, and molecular functions. The oxidation-reduction process (GO:0055114), carbohydrate catabolic process (GO:0016052), and transmembrane transport (GO:0055085) were the most significantly enriched GO terms in biological process, while the integral component of the membrane (GO:0016021)/extracellular region (GO:0005576) and the oxidoreductase activity (GO:0016491)/catalytic activity (GO:0003824)/binding (GO:0031177, GO:0019842, GO:0072341) were the most enriched GO terms in cellular component and molecular function (Figure 4A), respectively. Functional enrichment of the KEGG pathway of the upregulated and downregulated DEGs was also characterized. Enrichment analyses of the upregulated DGEs in the treatment of *A. oryzae* filtrates demonstrated that ABC transporters, genes involved in the primary metabolism (such as carbon metabolism and amino acid metabolism) and the secondary metabolism, were significantly enriched (Figure 4B). Additionally, the downregulated DGEs were significantly enriched in the metabolic pathway, antibiotic biosynthesis, and the precursors of secondary metabolites, such as terpenoid backbone and one-carbon pool (Figure 4C), indicating that the synthesis of secondary metabolites in *A. flavus* might be inhibited with the treatment of *A. oryzae* filtrates (Figure 4C).

### 2.5. Inhibition of Aflatoxin Biosynthesis Gene Cluster by the Treatment of A. oryzae Filtrates

**Figure 5:**
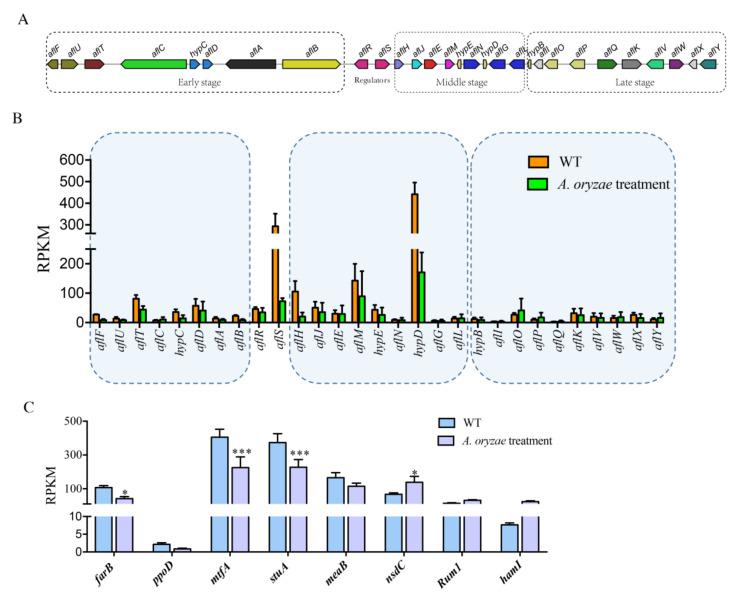
Effects of cell-free culture filtrate of *A. oryzae* Rib40 on the transcriptional expression of different stages of aflatoxin genes. (**A**) Aflatoxin gene cluster in *A. flavus* that was grouped into three stages according to the intermediates produced by their proteins in aflatoxin biosynthesis. (**B**) Expression levels (RPKM) of the 29 aflatoxin biosynthesis-related genes with/without *A. oryzae* Rib40 cell-free culture filtrate treatment. (**C**) Expression levels of known aflatoxin regulators. * statistically significant when compared to control, *p* < 0.05; *** statistically significant when compared to control, *p* < 0.001.

To better understand how *A. oryzae* filtrates affect AF biosynthesis in *A. flavus*, the expression levels of 29 genes that were required for the generation of AF within the biosynthesis gene cluster were observed and compared. The enzymatic reactions of AF are involved in three stages: early, middle, and late stages (Figure 5A). Here, we found that genes involved in early and middle stages of enzymatic reactions of AF were significantly downregulated in their transcription levels with the treatment of *A. oryzae* filtrates compared to those enzymes functioning at the late stage (Figure 5B). Interestingly, despite the pathway-specific regulatory transcription factor, AflR did not show a difference at its transcription expression level, and its partner, AflS, was transcriptionally inhibited under the treatment of *A. oryzae* filtrates (Figure 5B). In *A. flavus*, dozens of regulators have been reported to be participating in the regulation of AF production [20]. The transcriptome analysis demonstrated that a negative regulator of AF, NsdC [21], was activated under the treatment of *A. oryzae* filtrates (Figure 5C), while many of the positive regulators of AF, such as FarB [22], MtfA [23], and StuA [20], were significantly decreased in their expression levels (Figure 5C). 

### 2.6. The Effect of A. oryzae Filtrates on the Expression of Biosynthetic Gene Clusters (BGCs)

**Figure 6:**
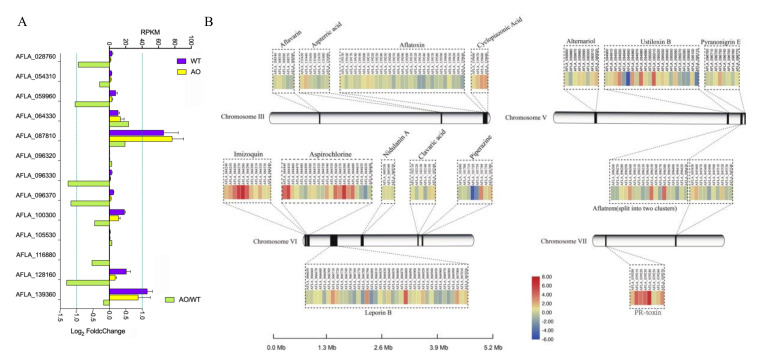
Effects of cell-free culture filtrate of *A. oryzae* Rib40 on gene expression of the biosynthesis gene clusters (BGCs) that are identified experimentally in *A. flavus*. (**A**) Relative expression levels of 13 transcriptional factors encoding genes identified among the BGCs in *A. flavus*. Top *X* axis indicates the RPKM values of the selected DEGs in WT (*A. flavus* wild type without any treatment) and AO (*A. flavus* wild type with *A. oryzae* cell-free culture filtrate treatment); bottom *X* axis presents the log2 FoldChange of the selected DEGs in AO when compared to WT. The dashed line indicates |log2 FoldChange| = 1. (**B**) Heat map and localization of experimentally identified BGCs in *A. flavus*. Heat map and chromosomal position of BGCs were visualized with TBtools [26]. To make the heat map, the original PRKM data were used and normalized with a log scale. A newly published *A. flavus* genome was used as a reference to visualize the chromosomal position of the BGCs [27].

The enrichment analysis demonstrated that DGEs involved in the metabolic pathway and the precursors of secondary metabolites were significantly downregulated in their expression (Figure 4). To determine whether *A. oryzae* filtrates have an impact on the other BGCs, we first analyzed the expression levels of 13 transcriptional factors (TFs) located in the predicted BGCs (Figure 6A). Former studies have revealed 74 BGCs, including the experimental identified SM in *A. flavus* [24,25], and here we found that only 13 transcriptional factors were located in the 11 predicted BGCs (Table S2), among which the expression levels of genes AFLA_128160 encoding a TF of cluster 5, AFLA_096330 and AFLA_096370 encoding two TFs of cluster 31 (expressing Aflatrem), and AFLA_059960 encoding a TF of cluster 71 were downregulated by more than twofold (Figure 6A). We further analyzed the expression data of the BGCs that are identified experimentally to produce SMs in *A. flauvus*. The results showed that most of the genes in the clusters of aflavarin and aspterric acid, together with aflatoxin localized in chromosome III, were transcriptionally reduced in their expressions. Most genes that are involved in the biosynthesis of leporin B, clavaric acid, and aflatrem were found to be depressed as well, while genes involved in the production of cyclopiazonic acid, imizoquin, and PR-toxin were significantly activated in response to *A. oryzae* filtrates (Figure 6B).

**Figure 7:**
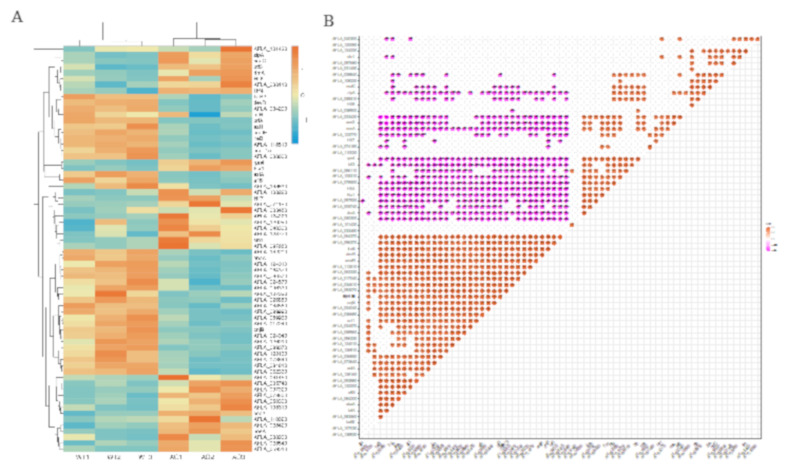
Effects of cell-free culture filtrate of *A. oryzae* Rib40 on the expression of the transcriptional factors encoding genes in *A. flavus*. (**A**) Clustering/heat map visualization of differentially expressed genes that encode transcriptional factors in *A. flavus*. (**B**) Correlation heat map of the DEGs that encode transcriptional factors in *A. flavus*. Data with a *p* value > 0.05 were excluded from the figure.

To better understand how *A. oryzae* filtrates affected the expression of BGCs transcriptionally, the expression levels of more than 200 TFs reported in *A. flavus* were analyzed. A total of 66 TFs were found to be expressed differently by more than twofold, among which half of them (33 for each) were significantly upregulated and downregulated, respectively (Figure 7A). To determine whether these TFs share a similarity in their expressing pattern, a correlation heat map was analyzed, and the result showed that AF-specific TF AflR was negatively correlated with AFLA_097920 (a putative C6 transcription factor). Intriguingly, another AF regulator AflS showed an expressed correlation with farB2 (a C6 transcription factor), AFLA_083560 (a putative C6 transcription factor), *brlA*, *abaA*, and AFLA_084200 (a putative C6 transcription factor) (Figure 7B).

### 2.7. Co-Regulated Gene Expression Network between Aflatoxigenic and Non-Aflatoxigenic Conditions

**Figure 8:**
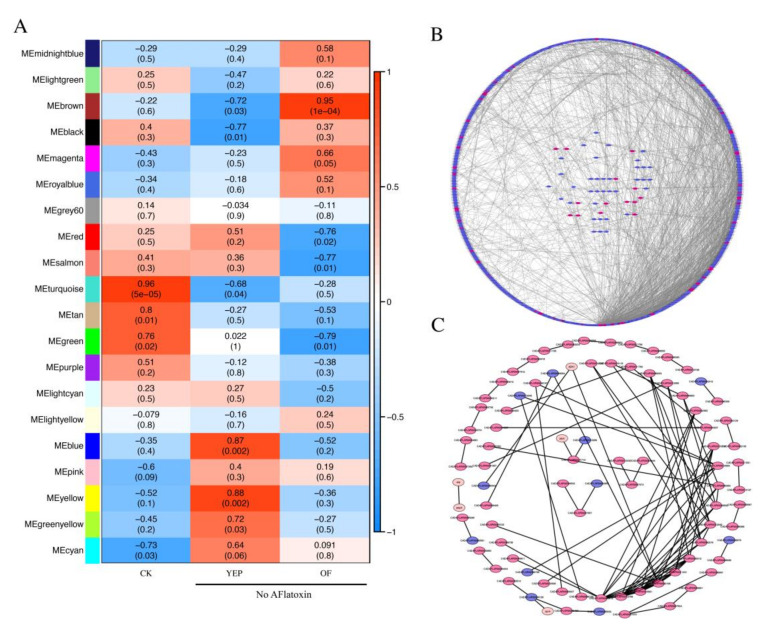
Analysis of the gene co-expression network between aflatoxigenic and non-aflatoxigenic conditions. (**A**) Association of the aflatoxin phenotype and consensus module eigengenes was performed with WGCNA analysis in aflatoxigenic and non-aflatoxigenic conditions. Gene network of the MEturquoise (**B**) and MEbrown (**C**) module was analyzed with the String online program (https://string-db.org) and further visualized with Cytoscape. Genes that were upregulated in a non-aflatoxigenic condition are indicated in red, while downregulated genes in a non-aflatoxigenic condition are shown in blue. CK, *A. flavus* wild type grown on GMM medium without any treatment; YEP, *A. flavus* wild type grown on an aflatoxin non-conducing YEP medium; AO, *A. flavus* wild type grown on a GMM medium with *A. oryzae* filtrate treatment. Values in (**A**) represent a positive correlation unless preceded by a minus, in which case values represent a negative correlation. The values in brackets indicate the *p* value.

Aflatoxins have been shown to regulate many environmental factors (such as pH and temperature) and culture conditions [28]. We previously reported that *A. flavus* failed to produce AF grown in YEP media [20]. To explore the general regulation mechanism of aflatoxins in *A. flavus*, an interaction network analysis between the aflatoxigenic and non-aflatoxigenic conditions (*A. oryzae* filtrate treatment and YEP-cultured media) was analyzed by WGCNA. The results demonstrated that the indicated RNA-seq data of YEP and with *A. oryzae* filtrate treatment (AO) or without *A. oryzae* filtrate treatment (CK) were clustered into 20 modules, which were marked with different colors (Figure 8A). The MEturquoise and MEbrown modules displayed the highest correction with the aflatoxin phenotype (R^2^ = 0.95 and R^2^ = 0.96, respectively). A total of 325 interacted genes of the MEturquoise module were utilized to generate the network (Figure 8B), while 87 genes (591 in total) of the MEbrown module were interacted to generate the co-expression network (Figure 8C). The information of the predicted interaction network is indicated in Table S4. The connection between the CADAFLAP gene number, from the String online program, and AFLA gene number is indicated in Table S5. Here, we found that three AF-related genes, including AFLA_139330 (*aflH*, CADAFLAP00007809), AFLA_139370 (*aflB*, CADAFLAP00007812), and AFLA_139400 (*hypC*, CADAFLAP00007815), which were all downregulated in the non-aflatoxigenic conditions, were identified in the MEturquoise module gene list. In the MEturquoise module, phosphoglycerate kinase PgkA (AFLA_069370, CADAFLAP00008361), GMP synthase (AFLA_137950, CADAFLAP00007672), pyruvate kinase (AFLA_087900, CADAFLAP00001568), nitrate reductase NiaD (AFLA_018810, CADAFLAP00003766), Cu,_Zn superoxide dismutase SOD1 (AFLA_099000, CADAFLAP00011638), and a ubiquitin-like modifier SUMO (AFLA_068730, CADAFLAP00008297) were among the core regulated network, which were all downregulated both in the *A. oryzae* filtrate treatment and YEP-cultured media (Figure 8B). Intriguingly, the most connected proteins, including citrate synthase Cit1 (AFLA_007020, CADAFLAP00010765), fumarate hydratase (AFLA_091270, CADAFLAP00008812), fructose-1,6-bisphosphatase Fbp1 (AFLA_027310, CADAFLAP00002106), and isocitrate lyase AcuD (AFLA_052400, CADAFLAP00011231) were identified among the MEbrown module gene list (Figure 8C). Importantly, the expression levels of their encoding genes, which are involved in the TCA (tricarboxylic acid) cycle and glycolysis, were all significantly reduced in the non-aflatoxigenic conditions. It was valuable to notice that the TCA cycle and glycolysis were potentially involved in aflatoxin biosynthesis here.

### 2.8. Inhibitory Regulation of Asexual Development Genes by the Treatment of A. oryzae Filtrates

**Figure 9:**
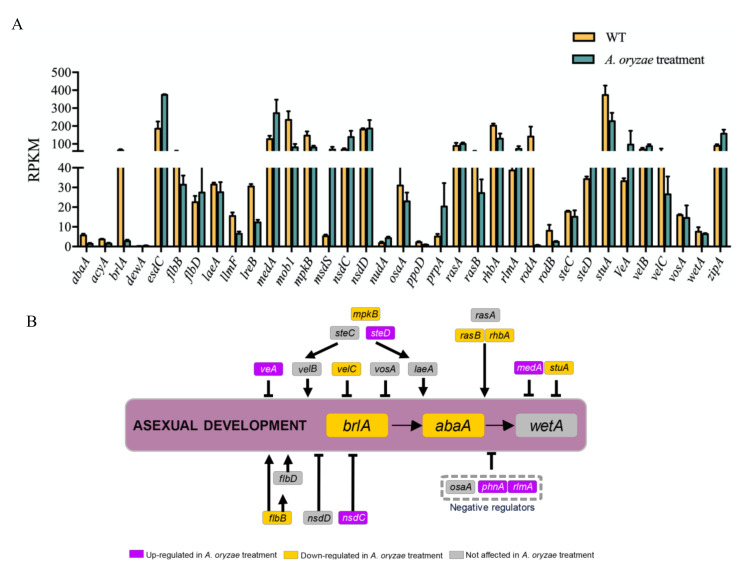
Effects of cell-free culture filtrate of *A. oryzae* Rib40 on the expression of genes that are involved in asexual development in *A. flavus*. (**A**) Expression levels (RPKM) of genes that are involved in asexual development with/without *A. oryzae* Rib40 cell-free culture filtrate treatment. (**B**) A schematic diagram of the regulatory model of asexual development. Genes with increased, decreased, and unaffected mRNA levels in the *A. oryzae* Rib40 cell-free culture filtrate are labeled in red, green, and gray, respectively.

Conidiation is one of the most important reproductive structures of *A. flavus* that can help it to spread in the environment and cause a series of contamination by this fungus. In this study, we found that *A. oryzae* filtrates could suppress asexual development of *A. flavus* (Figure 2). To determine the inhibitory regulation of *A. oryzae* filtrates on *A. flavus* reproduction, the transcriptional conditions of genes involved in asexual development were analyzed (Figure 9). Two important transcriptional factors that regulate asexual development, BrlA and AbaA, were found significantly decreased in their expression (Figure 9A,B), which might cause the downregulation of conidiation-specific genes, such as the conidial hydrophobins genes *rodA* and *rodB* (Figure 9A). In the regulatory networks, negative regulators of conidiation, such as Nsdc, MedA, PhnA, RlmA, and, in the velvet protein complex, VeA, were found remarkably increased in their expression at the transcriptional level in response to the *A. oryzae* filtrates (Figure 9B). Genes related to signal transduction, such as RAS small monomeric GTPase *rasA*, Rheb small monomeric GTPase coding gene *rhbA*, and MAP kinase coding gene *mpkB,* which have been reported to be involved in asexual development, were transcriptionally downregulated (Figure 9B). Taken together, the transcriptional data demonstrated the negative effects on asexual reproduction involved in the regulation of positive and negative regulators of conidiation in *A. flavus*.

## 3. Discussion

*A. flavus* is a saprophytic soil fungus that is notorious for its ability to colonize pre-harvest and post-harvest seed crops with one of the most toxic secondary metabolite aflatoxins, which has caused billion-dollar yield losses across the world [29]. This fungus is also an opportunistic pathogen of human and animals, causing aspergillosis diseases mostly due to the dispersion of the asexual spores in the air. This fungus is also hard to eliminate, both as a plant and human pathogen, due to its resistance to many common fungicides and a limited ability to apply fungicides to edible portions of plants or foodstuffs [30]. However, several different methods have been developed to combat this fungus and decrease the losses caused by its contamination and by mycotoxins [2,31,32,33]. 

Several studies indicating that the interaction of *A. niger* and other aspergilli with *A. flavus* is able to block aflatoxin biosynthesis have been reported [34,35,36]. In this study, we utilized *A. oryzae* and a non-aflatoxigenic *A. flavus* generated genetically to co-culture with wild-type *A. flavus*, and found that the production of AFB1 was remarkably inhibited (Figure 1). The cell-free culture filtrate of *A. oryzae* could more effectively inhibit the production of AFB1, which is consistent with the former study [34]. Early studies on *A. niger* demonstrated that, in a co-culture system with *A. flavus*, *A. niger* produced oxalic acid to suppress aflatoxin biosynthesis, partially due to the decrease in substrate pH levels of below 3.0 [37]. The antagonistic microbes against *A. flavus*, such as *Bacillus megaterium, Penicillium chrysogenum*, and *A. niger*, have been shown to secrete small antifungal peptides with low molecular weight that could inhibit AFB1 biosynthesis [38,39,40]. A similar result was found in this study. The cell-free culture filtrate was collected using a 1 KDa dialysis system to force metabolites and media through the filter. The inhibitory impact of the concentrated filtrates on *A. flavus* reproduction and AFB1 production suggested a presence of signal molecules in the culture filtrate that were able to restrict *A. flavus* development and AFB1 biosynthesis (Figure 2). Further research on the identification of the signal molecules produced by the non-aflatoxigenic aspergilli is needed to elucidate these findings further.

Nevertheless, the mechanism of the inhibition of AFB1 biosynthesis by these aspergilli remains unclear. In this study, to analyze the inhibitory mechanism of *A. flavus* treated with fermenting cultures, transcriptome sequencing was performed, which demonstrated that genes involved in the early and middle stages of enzymatic reactions of AFB1 were significantly decreased in their expression levels with the treatment of *A. oryzae* filtrates (Figure 5). One of the AF pathway-specific regulator AflS was found to be transcriptionally downregulated in response to the treatment of *A. oryzae* filtrates (Figure 5). Some other transcription factors (TFs) that have been reported to be involved in the regulation of AF production were found to be expressed differently in this study. For instance, a negative regulator of AF, NsdC, was found to be upregulated under the treatment of *A. oryzae* filtrates (Figure 5), while the positive regulators of AF, such as FarB, MtfA, and StuA, were significantly decreased in their expression levels (Figure 5). The differential expression of these TFs could potentially inhibit the activation of the BGC of AFB1, leading to a reduction of AF biosynthesis. In addition to AFB1, the *A. oryzae* filtrates were found to have a similar inhibitory impact on many of the biosynthesis gene clusters in *A. flavus*. Most genes that are involved in biosynthesis of aflavarin, leporin B, and aflatrem were found to be significantly downregulated (Figure 6), while genes involved in cyclopiazonic acid, imizoquin, and PR toxins were significantly activated in response to *A. oryzae* filtrates (Figure 6). In *A. nidulans*, the antifungal protein PAF isolated from *P. chrysogenumare* was shown to inhibit the growth of *A. nidulans* by interfering with PCK/MPK and cAMP/PKA signals [41]. Here, we also found that most of the heat shock proteins were transcriptionally activated (Figure S1), while most of the G protein-coupled receptors were found to be downregulated in response to the treatment of *A. oryzae* filtrates (Figure S1), which might lead to an inactivation of their downstream signaling pathway. Intriguingly, our WGCNA results revealed that genes involved in the TCA cycle and glycolysis were significantly downregulated in the non-aflatoxigenic conditions (Figure 8C). It has been demonstrated that, in addition to providing energy, the TCA cycle and glycolysis are the major sources of precursors of secondary metabolites in filamentous fungi [29]. Thus, blocking the TCA cycle and glycolysis can potentially inhibit biosynthesis of many important secondary metabolites, including aflatoxins.

The inhibition of the secreted proteins produced by aspergilli on *A. flavus* growth has been reported in many studies [35,42]. Here, we found that the asexual development of *A. flavus* was obviously suppressed by *A. oryzae* filtrates (Figure 2). In *Aspergillus* molds, asexual development is regulated by the BrlA > AbaA > WetA transcriptional cascade [24]. Our comparative transcriptomics revealed that *brlA* and *abaA* (but not *wetA*) were found to be significantly decreased in their expression (Figure 9), while some other negative regulators of conidiation, such as velvet protein complex, VeA, Nsdc, MedA, PhnA, and RlmA, were transcriptionally activated in response to the *A. oryzae* filtrates (Figure 9), which might together lead to the downregulation of conidiation-specific genes, such as the conidial hydrophobins genes *rodA* and *rodB* (Figure 9). Intriguingly, the C2H2-type conidiation transcription factor BrlA that is required for asexual development in *Aspergillus* molds was found to be a key regulator of BGCs and secondary metabolites that are regulated by LaeA in an epigenetic manner in *A. fumigatus* [43]. Although *laeA* expressed no difference in response to the *A. oryzae* filtrate treatment, *brlA* was found to be remarkably downregulated, which might partially explain why many of the BGCs were suppressed in response to *A. oryzae* filtrate treatment (Figure 6).

## 4. Materials and Methods 

### 4.1. Strains and Culture Conditions

The *Aspergillus* strains used in this study are indicated in Table 1, among which *A. flavus* NRRL 3357 is an aflatoxigenic wild-type stain, *A. oryzae* RIB40, and TSJ-1 (an MAPK kinase SskB-null mutant of *A. flavus* that fails to produce aflatoxins) are served as non-aflatoxigenic aspergilli. All strains were inoculated on potato dextrose agar (PDA) medium (20% potato, 2% dextrose, and 1.5% agar) and cultured in the dark at 30 °C for 7 days. Spores were collected from individual cultures on PDA with 0.001% Tween-20 solution after filtering mycelia with four layers of wipe paper. The spores were quantified hemocytometrically, and the numbers were adjusted to 10^6^ conidia/mL with distilled water for further study.

### 4.2. Effect of Co-Cultivation of Non-Aflatoxigenic Aspergilli on the Growth of A. flavus and AFB1 Production

A series of dilution of non-aflatoxigenic *Aspergillus* strains’ spores (10^3^, 10^5^, and 10^6^ spores/mL) were, respectively, added to a 50-mL YES (yeast extract and sucrose) medium containing 1 mL of 1 × 10^6^ conidia/mL suspensions of *A. flavus* NRRL3357, and incubated in the dark at 30 °C for 9 days with shaking at 150 rpm. The control without additional spores of non-aflatoxigenic *Aspergillus* was performed under the same conditions. The experiments were conducted with three replicates for all treatments. The AFB1 production in the culture medium was assayed after 3 days and after 9 days of incubation. Aflatoxins were then extracted according to a previously described method [44]. Briefly, 500 µL of culture medium were used for AF extraction with chloroform and thin-layer chromatography (TLC) was performed to analyze AFB1 production.

### 4.3. Effect of the Culture Filtrate of Non-Aflatoxigenic Aspergilli Strains on A. flavus Development and AFB1 Production

The analysis of the cell-free culture filtrate of non-aflatoxigenic aspergilli on wild-type *A. flavus* development and AF biosynthesis was performed according to the previously reported method with minor modification [35]. A total number of 10^7^ *A. oryzae*/TSJ-1 conidia were added to 500 mL of potato dextrose broth (PDB) and cultured at 30 °C for 5 days with shaking at 180 rpm.

The cell-free culture filtrate was collected by filtering hyphae. To concentrate the culture filtrates, a 1 KDa dialysis bag (Sangon Biotech, Shanghai, China) with the filtrates was placed over a bed of polyethylene glycol (PEG) and covered with more PEG, and the compounds with a weight less than 1 KDa were dialyzed from the bag. The concentrated culture filtrates were filtered with a 0.2-μm-diameter filter for future use. To assay the effect of concentrated cell-free culture filtrates of non-aflatoxigenic aspergilli on *A. flavus* development, 2 μL of 10^6^ conidia/mL of wild-type *A. flavus* were spotted onto solidified PDA plates containing 4% or 8% of the concentrated filtrates, and were incubated for 7 days at 30 °C in the darkness. The PDA plates without supplemental concentrated filtrates were used as a control. The diameter of the colony and the spore’s production of *A. flavus* were measured after 7 days of inoculation.

All treatments were tested in three replicates. To assay its influence on AFB1 biosynthesis, 1 mL of 1 × 10^6^ conidia/mL suspensions of *A. flavus* NRRL3357 was added to 50 mL of YES with a supplementation of 8% of the concentrated filtrates, and were incubated in the dark at 30 °C for 4 days with shaking at 150 rpm. The extraction and detection of AFB1 are described above. For quantitative analysis of AF production from the TLC result, the ImageJ software was used.

### 4.4. RNA Isolation

A total number of 10^7^ *A. flavus* wild-type conidia were added to 50 mL of glucose minimal medium (GMM) and cultured at 30 °C for 24 h with shaking at 180 rpm, and 8% of the concentrated filtrates of *A. oryzae* RIB40 were then added to the medium with continued culturing at 30 °C for 24 h. The experiments were conducted with three replicates. The mycelia were harvested and frozen in liquid nitrogen, and were lyophilized for 24 h. Total RNA was extracted with Trizol (Invitrogen, Carlsbad, CA, USA), according to the manufacturer’s protocol. The quality and integrity of RNA samples were determined using a Nanodrop and an Agilent 2100 bioanalyzer (Agilent Technologies, Palo Alto, CA, USA), respectively. The quantity of RNA samples was further measured with a Qubit RNA assay kit (Life Invitrogen, Carlsbad, CA, USA).

### 4.5. RNA-Seq and Enrichment Analysis of Differentially Expressed Genes

The total RNA of three biological replicates for the treatment of *A. oryzae* concentrated filtrates and control (without treatment) was sequenced. A standard protocol from Illumina Inc. (San Diego, CA, USA) and sequencing on a HiSeq 2000 platform (Berry Genomics, Beijing, China) were used for preparing the libraries. The sequenced clean reads were mapped against predicted transcripts of the *A. flavus* NRRL 3357 genome using hisat2 [45] and Samtools [46]. 

Transcript abundance was estimated using the Featurecount package [47]. Differentially expressed genes (DEGs) were screened by a comparison of *A. oryzae* concentrated filtrate treatment groups with control groups and then analyzed with DESeq package using RStudio software, and both a 2-fold change cut-off and an adjusted *p*-value of ≤0.05 were established as thresholds. The enriched GO terms and KEGG pathways of DEGs were analyzed with OmicShare Tools (https://www.omicshare.com/tools/).

### 4.6. Weighted Correlation Network Analysis (WGCNA) of Co-Expression Gene Network 

The co-expression gene network was analyzed by a WGCNA R package [48]. The RPKM (reads per kilobase per million mapped reads) of all *A. flavus* genes were used as input for the WGCNA, and an “unsigned” type was applied to create the network. The weighted matrix of pair-wise connection strengths (module) was built, and genes were grouped into modules by hierarchical clustering. The power β with a value of 9 was used to calculate the correlation coefficients. The selective eigengenes module was analyzed with the String online program (https://string-db.org) and further visualized with Cytoscape.

### 4.7. Statistical Analysis

The results were analyzed using one-way analysis of variance (ANOVA) and Student’s *t*-test as appropriate with GraphPad Prism 7 software (San Diego, CA, USA) for significance analysis of multiple comparisons and comparison of two different groups, respectively. Each treatment consisted of three replicates and was expressed as mean ± SD (standard deviation).

## 5. Conclusions

Our data showed that *A. oryzae* and the non-aflatoxigenic *Aspergillus* have potential biocontrol activity to inhibit aflatoxin biosynthesis and *A. flavus* asexual development. Comparative transcriptomics further revealed the inhibitory mechanism of aflatoxin metabolism and asexual development in *A. flavus*. This study may potentially provide the antifungal agent against *A. flavus* and aflatoxins, which are safe for agricultural harvest.

## Figures and Tables

**Table 1:** *Aspergillus* strains used in this study.

Name of Strain	Genotype	Source
NRRL 3357	*A. flavus* Wild type	Keller lab [3]
RIB40	*A. oryzae* Wild type	Keller lab [3]
TSJ-1	Δ*ku70,* Δ*sskB*:*AfupyrG*	Wang lab

## Supplementary Materials

Supplementary materials can be found at https://www.mdpi.com/1422-0067/21/19/6994/s1.

## Abbreviations

AF	Aflatoxin
DEGs	differentially expressed genes
RPKM	Reads Per Kilobase per Million mapped reads
GO	Gene Ontology
KEGG	Kyoto Encyclopedia of Genes and Genomes
WGCNA	Weighted correlation network analysis
