# Supplementary material for: Transcriptome Sequencing Revealed an Inhibitory Mechanism of Aspergillus flavus Asexual Development and Aflatoxin Metabolism by Soy-Fermenting Non-Aflatoxigenic Aspergillus
Source: Int J Mol Sci. 2020 Sep 23;21(19):6994. doi: 10.3390/ijms21196994 (PMC7583960; doi:10.3390/ijms21196994)
Supplement: Supplementary file 1 [file ijms-21-06994-s001.zip › ijms-898523-supplementary/Supporting information/1 Supporting information.docx]

**Supporting information**

Figure S1. Relative expression level of the selective DEGs in *A. flavus*. (A) genes encoding G protein-coupled receptor, (B) genes encoding heat shock proteins and alternative oxidase. Left Y axis indicates the RPKM values of the selected DEGs in WT (*A. flavus* wild type without any treatment) and AO (*A. flavus* wild type with *A. oryzae* cell-free culture filtrate treatment); Right Y axis presents the log2 FoldChange of the selected DEGs in AO when compared to WT. The green dashed line indicates |log2 FoldChange| = 1.

Table S1. Summary of sequencing data and mapped results in this study.

| sample | Duplicates | Raw reads | Raw base | Clean reads | Clean base | Clean reads ratio (%) | Q20(%) | Q30(%) | Mapped (%) |
| --- | --- | --- | --- | --- | --- | --- | --- | --- | --- |
| F2-1 | 1 | 20,924,021 | 6,277,206,300 | 20,345,729 | 6,103,718,700 | 97.24 | 96.23 | 90.96 | 83.85% |
| F2-2 | 2 | 23,341,436 | 7,002,430,800 | 22,806,785 | 6,842,035,500 | 97.71 | 96.08 | 90.72 | 95.65% |
| F2-3 | 3 | 21,204,189 | 6,361,256,700 | 20,283,623 | 6,085,086,900 | 95.66 | 96.19 | 91.18 | 95.28% |
| OF1 | 1 | 22,965,987 | 6,889,796,100 | 22,356,498 | 6,706,949,400 | 97.35 | 96.32 | 91.225 | 93.65% |
| OF2 | 2 | 19,866,024 | 5,959,807,200 | 19,033,799 | 5,710,139,700 | 95.81 | 96.12 | 90.66 | 91.73% |
| OF3 | 3 | 24,151,283 | 7,245,384,900 | 23,563,069 | 7,068,920,700 | 97.56 | 96.30 | 91.055 | 92.11% |

Table S2. Expression data of 13 transcriptional factors encoding genes identified among the BGCs in *A. flavus*.

| Cluster | Gene | RPKM | | | | | | log2FoldChange  (AO/WT) | Chromosome |
| --- | --- | --- | --- | --- | --- | --- | --- | --- | --- |
|  |  | WT1 | WT2 | WT3 | AO1 | AO2 | AO3 |  |  |
| 5 | AFLA_128160 | 26.19 | 19.59 | 15.99 | 6.88 | 5.47 | 8.76 | -1.31 | II |
| 8 | AFLA_054310 | 3.11 | 2.34 | 2.09 | 1.12 | 2.08 | 2.00 | -0.30 | I |
| 18 | AFLA_087810(metZ) | 85.47 | 63.07 | 50.11 | 91.96 | 73.42 | 64.37 | 0.47 | I |
| 21 | AFLA_116880 | 0.25 | 0.18 | 0.18 | 0.13 | 0.08 | 0.13 | -0.53 | VIII |
| 31 | AFLA_096320 | 0.42 | 0.38 | 0.23 | 0.07 | 0.43 | 0.42 | 0.07 | V |
| 31 | AFLA_096330 | 3.05 | 3.19 | 1.75 | 0.65 | 0.61 | 1.57 | -1.27 | V |
| 31 | AFLA_096370 | 4.63 | 5.55 | 4.78 | 1.47 | 1.99 | 2.16 | -1.18 | V |
| 40 | AFLA_100300 | 17.62 | 19.73 | 17.50 | 12.43 | 8.99 | 12.29 | -0.45 | VI |
| 44 | AFLA_064330(fmpR) | 9.32 | 12.73 | 9.85 | 8.79 | 17.93 | 13.74 | 0.59 | VI |
| 59 | AFLA_139360(aflR) | 44.48 | 41.00 | 53.20 | 20.07 | 35.47 | 49.81 | -0.18 | III |
| 63 | AFLA_028760 | 2.51 | 4.56 | 2.67 | 1.03 | 1.28 | 1.99 | -0.95 | II |
| 66 | AFLA_105530 | 1.23 | 0.56 | 0.43 | 0.65 | 0.46 | 0.85 | 0.07 | III |
| 71 | AFLA_059960 | 7.98 | 9.44 | 5.53 | 2.65 | 2.58 | 4.16 | -1.05 | V |
